# Mutations in the *Drosophila* ortholog of the vertebrate Golgi pH regulator (GPHR) protein disturb endoplasmic reticulum and Golgi organization and affect systemic growth

**DOI:** 10.1242/bio.20137187

**Published:** 2013-12-06

**Authors:** Bernard Charroux, Julien Royet

**Affiliations:** Aix-Marseille Université, CNRS, Institut de Biologie du Développement de Marseille-Luminy UMR 7288, F-13288 Marseille, France

**Keywords:** GPHR, *Drosophila*, Golgi, Growth control, Endoplasmic reticulum

## Abstract

Sorting of secretory cargo and retrieval of components of the biosynthetic pathway occur in organelles such as the Golgi apparatus, the endoplasmic reticulum and the endosomes. In order to perform their functions in protein sorting, these organelles require a weakly acidified lumen. In vitro data have shown that Golgi luminal pH is in part regulated by an anion channel called Golgi pH Regulator (GPHR). Mammalian cells carrying a mutated GPHR version present an increased luminal pH leading to delayed protein transport, impaired glycosylation and Golgi disorganization. Using *Drosophila* as a model system, we present here the first phenotypic consequences, at the organism level, of a complete lack of GPHR function. We show that, although all individuals carrying complete loss-of-function mutations in the *dGPHR* gene can go through embryonic development, most of them die at late larval stages. The *dGPHR* mutations are, however, sublethal and can therefore generate escapers that are smaller than controls. Using cellular and molecular readouts, we demonstrate that the effects of *dGPHR* mutation on larval growth are not due to Insulin signaling pathway impairment and can be rescued by providing dGPHR in only some of the larval tissues. We reveal that, although functionally exchangeable, the invertebrate and vertebrate GPHRs display not completely overlapping sub-cellular localization. Whereas the mammalian GPHR is a Golgi-only associated protein whose inactivation disturbs the Golgi apparatus, our data suggest that dGPHR is expressed in both the ER and the Golgi and that dGPHR mutant flies have defects in both organelles that lead to a defective secretory pathway.

## Introduction

The main function of the Golgi apparatus is to sort molecules that are transported through this organelle en route to the plasma membrane, the extracellular medium and the endosomal/lysosomal compartments. As for each individual organelle, the function of the Golgi apparatus depends on the establishment and stringent maintenance of a distinct pH (Casey et al., 2010). In mammalian cells, luminal acidification of the Golgi apparatus is essential for its function and to maintain cellular homeostasis (Weisz, 2003). Indeed, when the acidic luminal pH is artificially alkalinized, the trafficking, processing and glycosylation of cargo proteins and lipids are impaired. As a result, some proteins become misrouted and the morphological integrity of the Golgi is compromised (Axelsson et al., 2001; Rivinoja et al., 2006). Consequently, mutations that alter Golgi luminal pH have been shown to perturb cell metabolism leading to congenital diseases and cancer (Weisz, 2003). The acidic pH of organelles, including the Golgi, is regulated by a balance between the proton pump V-ATPase, which is the sole proton delivery source, and the counterion channel GPHR (Moriyama and Nelson, 1989; Nishi and Forgac, 2002; Schapiro and Grinstein, 2000; Wu et al., 2001). This voltage dependent anion channel dissipate the membrane potential formed by the proton influx allowing proton pump to transfer more protons into the Golgi lumen and therefore facilitating Golgi luminal acidification and hence Golgi function. Cells in which GPHR is inactive show elevated luminal pH of the Golgi but not of the endosomal/lysosomal compartments (Maeda et al., 2008). Using a keratinocyte-specific GPHR-knockout mice model, Medea and collaborators have recently shown that GPHR is essential for the homeostasis of the epidermis including the formation of lamellar bodies and for establishing its barrier function (Tarutani et al., 2012).

*Drosophila* has recently been established as a good alternative model system to study the Golgi apparatus that shares many morphological and functional similarities with the mammalian one (Kondylis and Rabouille, 2009; Kondylis et al., 2007; Sisson et al., 2000). *Drosophila* is also very suitable to study the anatomical and physiological consequences associated with gene inactivation at the organism level. We present here the molecular and phenotypic characterization of null loss-of-function alleles of the *Drosophila* ortholog of the mammalian GPHR anion channel. We show that the complete inactivation of dGPHR is not always associated with fly lethality but dramatically impairs its developmental growth independently of the Insulin signaling (IS) pathway. We also demonstrate that although dGPHR is indeed the functional ortholog of the human GPHR, its subcellular localization and function differ from the mammalian protein. Whereas mammalian GPHR is a Golgi-only protein require for pH maintenance, the *Drosophila* ortholog co-localized with markers of both endoplasmic reticulum and Golgi and is required for correct organization of both organelles.

## Materials and Methods

### *Drosophila* melanogaster strains and maintenance

The following strains were used in this work: *dGPHR^k34^* (this work), *dGPHR^LL03674^* (Kyoto#140780), *elav-Gal4* (BL#458), *c601-Gal4* (BL#30844), *Mef2-Gal4* (BL#27390), *daughterless-Gal4* (BL#5460), *UAS-Galt::RFP* (BL#30907), *UAS-KDEL::RFP* (BL#30909), *UAS-myrRFP* (BL#7118), *UAS-secGFP* (Entchev et al., 2000) and *chico^1^* (BL#10738). The *UAS-dGPHR*, *UAS-dGPHR::HA*, *UAS-huGPHR*, *UAS-huGPHR::HA* and *UAS-hamsGPHR* flies were obtained by P element mediated insertion of *pUAST* constructs containing full length cDNA coding for dGPHR, dGPHR::HA, huGPHR, huGPHR::HA and hamsGPHR, respectively (molecular details available upon request).

Flies were grown at 25°C on a yeast/cornmeal medium. For 1 litre of food, 8.2 g of agar (VWR, cat. no. 20768.361), 80 g of cornmeal flour (Westhove, Farigel maize H1) and 80 g of yeast extract (VWR, cat. no. 24979.413) were cooked for 10 min in boiling water; 5.2 g of methylparaben sodium salt (Merck, cat. no. 106756) and 4 ml of 99% propionic acid (Carloerba, cat. no. 409553) was added when the food had cooled down.

### Measurement of weight, larval size, pupariation, and adult emergence

Adult size was estimated based on the weight of 3-day-old flies. The weight of multiple replicates (minimum of three) of a pool of five females or males was calculated using a precision balance (Mettler Toledo, AG245). Larval size was estimated by collecting and freezing larvae (n>20) twice a day (morning and evening) after an initial 3 hr period of egg deposition. Larvae were frozen and mounted in 80% glycerol in PBS. Pictures were taken on a black background using a ProgResC5 CCD camera (JenOptik) mounted on a stereomicroscope. The body surface of each larva was calculated using ImageJ. Masks covering the surface of the larvae were generated using the threshold tool. Surface values were displayed in pixels. To examine the time of pupariation and adult emergence, 40 eggs were collected for 5 hr, and the number of new pupa or adults was counted every 24 hr.

### Determination of wing cell size and cell number

For each genotype, eight to ten wings were photographed. Cell size and total cell numbers in the wing were estimated by counting the number of wing hairs within the constant pixel area to determine relative pixel area per cell. Relative cell number was estimated by dividing the total wing pixel area by the pixel area per cell.

### Clone induction

Mitotic clones were generated by FLP-mediated mitotic recombination (Xu and Rubin, 1993). Induction of *dGFPHR^k34^* mutant clones in wing imaginal discs was obtained by crossing females *ywhspfl*; *ubi-GFP FRTG13/ubi-GFP FRTG13* to males *dGFPHR^k34^ FRTG13/CyO Dfd::GFP*. Larvae of the progeny were heat shocked at L2 stage (48–72 hr after egg deposition, AED) and observed 24 hr later. Mutant clones were identified by absence of GFP.

Generation of MARCM (Mosaic analysis with a repressible cell marker) clones in salivary glands was performed by crossing MARCM virgin females of genotype *ywhsflp*, *UAS-GFP*, *act-Gal4*; *Tub-Gal80 FRTG13* en masse to the *dGFPHR^k34^ FRTG13/CyO Dfd::GFP* line. Resulting embryos were submitted to a heat shock 4–6 hr AED for 1 hr at 38°C in a circulating water bath, and kept at 25°C until larvae reached mid late third instar (120 hr AED).

### Imaging

Larval tissue were dissected in PBS and fixed for 20 min in 4% paraformaldehyde on ice. After several rinses in PBT (PBS + 0.1% Triton X-100), the tissues were mounted in Vectashield (Vector Laboratories) fluorescent mounting medium, with DAPI. Phalloidin staining of F-actin was performed by incubating wing disc with Alexa Phalloidin 546 (Invitrogen) at 1:250 in PBT 30 min before fixation. For antibody staining, larval tissue were dissected in PBS and fixed for 20 min in 4% paraformaldehyde on ice. After several rinses in PBT (PBS + 0.1% Triton X-100), they were blocked for 1 hr in PBT–3% BSA at 4°C and then incubated in antibody at the appropriate dilution (anti-HA 12CA5 1:500, Abcam; and rabbit anti-RFP 1:500, Rockland) in PBT-BSA 3% overnight at 4°C. Several rinses in PBT were followed by a 2 hr incubation in secondary antibody at RT (Alexa Fluor 488 donkey anti-mouse and Alexa Fluor 555 donkey anti-rabbit diluted 1:500, Molecular Probes), then 5 rinses in PBT. The tissues were finally mounted in Vectashield (Vector Laboratories). Images were captured with a LSM 780 Zeiss confocal microscope.

### Electronic microscopy

For electron microscopic sections, third instar salivary glands were dissected and fixed at RT in 4% PFA and 2% glutaraldehyde in 0.12 M sodium cacodylate buffer at pH 7.4 for 1 hr. The salivary glands were then washed for 3610 min in 0.12 M sodium cacodylate buffer, post-fixed in 2% OsO_4_ in 0.12 M sodium cacodylate buffer for 1 hr and washed again 3×10 min. Samples were subsequently dehydrated through series of ethanol gradients and infiltrated with propylene oxide, embedded in epoxy resin (Fluka, Sigma) and polymerized at 80°C. Ultrathin (80 nm) plastic sections were cut using a Leica UltraCut microtome with a diamond Diatome knife and post-stained with 2% uranyl acetate, followed by treatment with Reynolds' lead citrate, and stabilized for transmission EM by carbon coating. Examination was performed with a Zeiss Leo 912 microscope at 100 kV. Images were captured using a Gatan 792 Bioscan camera using Digital Micrograph software.

### Quantitative real-time PCR

Quantitative real-time PCR, TaqMan, and SYBR Green analysis were performed as previously described (Charroux and Royet, 2009). Primer information can be obtained upon request. The amount of mRNA detected was normalized to control *rp49* mRNA values. Normalized data were used to quantify the relative levels of a given mRNA according to cycling threshold analysis (ΔCt).

### Mouth hook contractions

Larvae were rinse 30 seconds in PBS and transferred to agar plate and quantified for the frequency of mouth-hook contraction by individual larva at 22°C. Typically, the assay time was 20 min. The contraction frequencies of individual larva remained consistent throughout the assay.

## Results

### *CG8090* encodes the invertebrate ortholog of GPHR

**Fig. 1.**
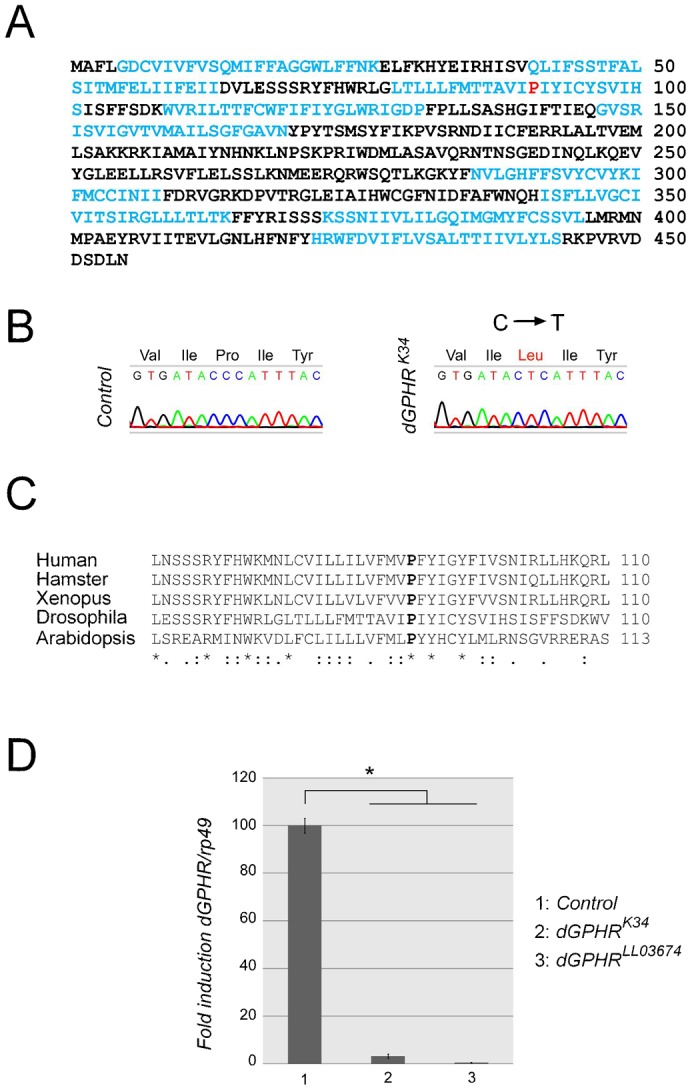
Molecular characterization of the *dGPHR^k34^* mutation. (A) Amino acid sequence of the *Drosophila* GPHR protein showing in black the putative transmembrane domains. (B) Sequencing of GPHR DNA from *dGPHR^k34^* mutant flies reveals the presence of a missense mutation (CCC→CTC) in the codon for Pro 91. (C) Protein sequence comparison showing the evolutionary conservation of the third transmembrane domain and the Pro 91 of the GPHR proteins. (D) qRT-PCR showing the effect of two independent mutations on dGPHR mRNA levels.

Following an EMS mutagenesis, we isolated an allele carrying a point mutation in a gene referred as *CG8090* in *Flybase* (http://flybase.org). Protein prediction analysis indicates that *CG8090* transcript encodes the *Drosophila* ortholog of vertebrate GPHR (*dGPHR*), a ubiquitously expressed protein containing nine transmembrane domains whose AA sequence is conserved among vertebrates, insects and plants (Maeda et al., 2008) and Fig. 1A. Genomic DNA sequencing of the EMS *dGPHR* allele (called *dGPHR^k34^*) revealed the presence of a missense mutation in the *dGPHR* coding region that substitute an highly conserved Proline into a Lysine in the third transmembrane domain of the protein (Fig. 1B,C). A second non-complementing allele of *dGPHR* (*dGPHR^LL03674^*) corresponding to a *PiggyBac* transposon insertion into the fifth intron of *dGPHR* was also used in this study. We found that both *dGPHR^k34^* and *dGPHR^LL03674^* mutations affect *dGPHR* mRNA stability as demonstrated by qRT-PCR quantification (Fig. 1D).

### *dGPHR* mutant escapers develop into dwarf flies

**Fig. 2.**
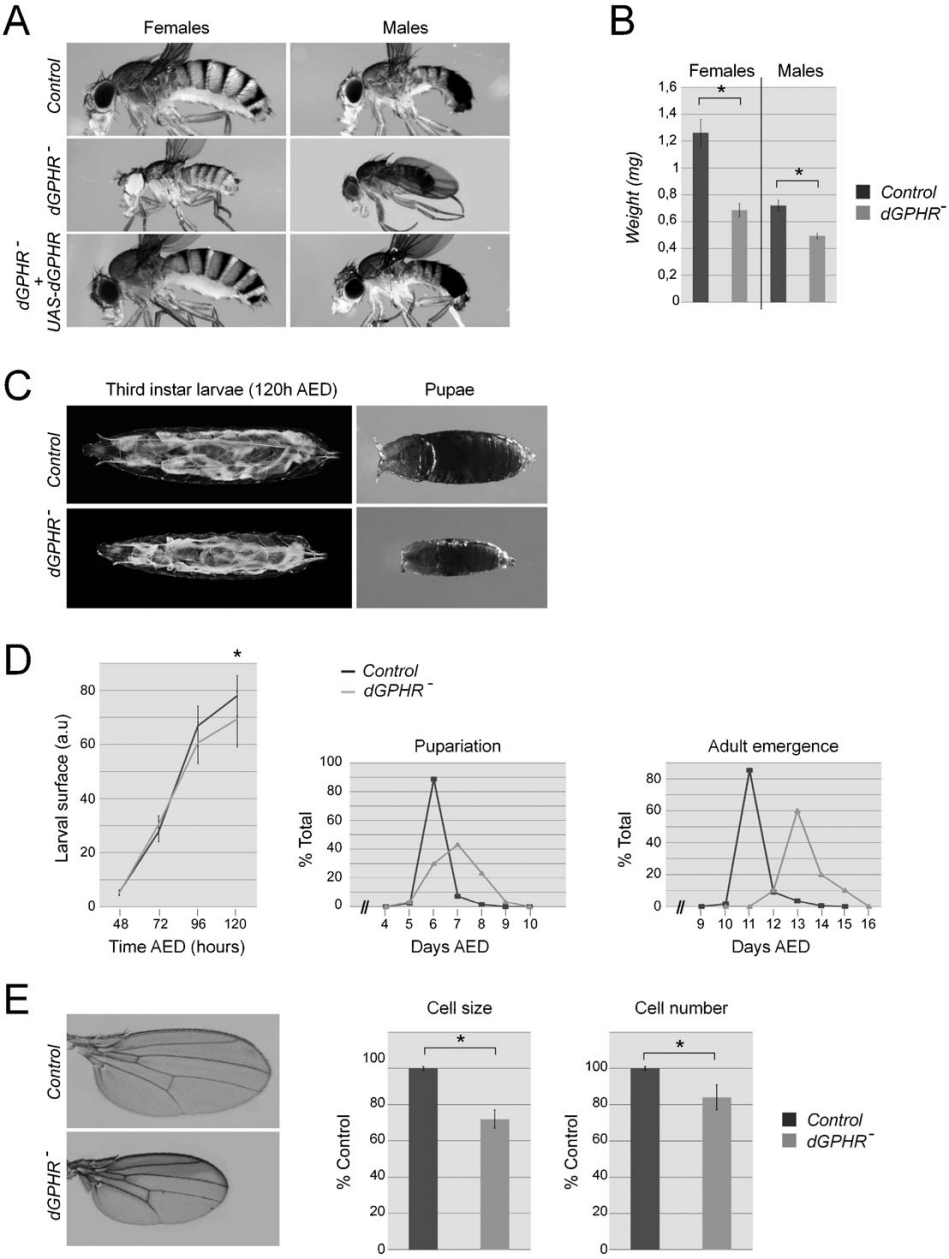
Phenotypic characterization of the *dGPHR* mutants. (A–C) dGPHR mutations affect the size of larvae, pupae and adults. For panel B, the weights of 30 adults male and female were measured. (D) Larval surface over time, pupariation and adult emergence period of control and *dGPHR^−^* grown in rich diet medium. (E) Adult wing size, wing cell size and numbers in a given area of wild-type and *dGPHR* mutant animals. Values indicated by * are statistically significant (p<0.05).

Phenotypic analysis of *dGPHR^k34^* and *dGPHR^LL03674^* homozygous mutants and transheterozygous allelic combinations points to an essential role of *dGPHR* in regulating developmental growth and body size of *Drosophila* (Fig. 2). When raised in standard medium conditions, most *dGPHR* mutants die as late larvae. However, some individuals go through the pupal stage and give rise to pharate that are significantly smaller and lighter than control animals (Fig. 2A,B). A precisely timed quantification of larval growth indicates that although *dGPHR* mutant and wild-type larvae grow equally until late mid L3 stage (96 hr AED), *dGPHR* mutant larvae show growth deceleration at late L3 (between 96 hr and 120 hr AED) (Fig. 2D). By late L3 and pupal stages, size differences between wild-type and mutant siblings are obvious (Fig. 2C). In addition, the onset of both pupariation and adult emergence are delayed in *dGPHR* null flies compared to wild-type controls (Fig. 2D). Thus, the smaller body size of *dGPHR* mutants is likely to be caused by slower growth rate during the late larval period rather than by precocious pupariation. Note that few *dGPHR* mutants can reach pupal (20%±7 SD of expected pupae) and adult (5%±2 SD of expected adults) stages.

To test whether this overall size reduction was due to a decrease in cell size, cell number or both, we quantified these parameters in adult wing blades. As shown in Fig. 2E, both cell size and cell number were reduced in *dGPHR* mutant when compared to controls. The similar penetrance and strength of the phenotype observed between *dGPHR* homozygous mutants and *dGPHR^k34^*/*Df(2R)ED2426* or *dGPHR^LL03674^*/*Df(2R)ED2426* allelic combinations (data not shown) demonstrate that *dGPHR^k34^* and *dGPHR^LL03674^* are complete loss-of-function alleles as far as growth control is concerned.

### *dGPHR* is not a component of the Insulin pathway

**Fig. 3.**
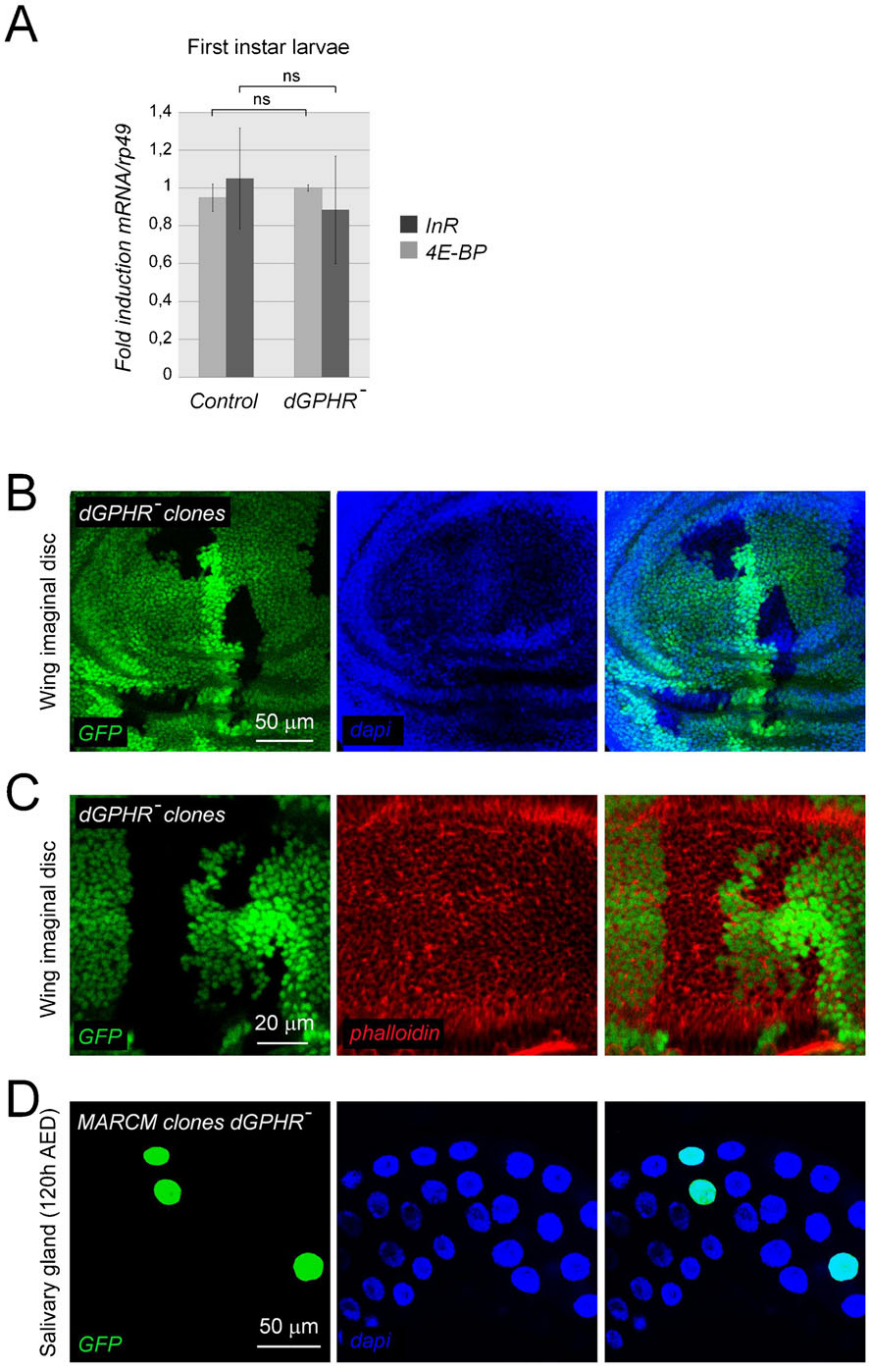
*dGPHR* mutations do not impact the Insulin pathway signaling. (A) mRNA quantification by qRT-PCR of two transcriptional targets of the IS pathway. *dGPHR* mutations do not affect *InR* and *4E-BP* transcription. (B–D) Confocal microscope section of a third instar wing imaginal disc containing mitotic clones of *dGPHR^k34^* mutant cells (absence of green staining), and wild-type (bright green staining) sister clones. (B) *dGPHR* mutant clones and wild-type sister clones have similar size. (C) Size of wild-type and *dGPHR* mutant cells labeled with phalloidin is similar. (D) Nuclei size of salivary gland cells is not affected by a mutation in the *dGPHR*. Scale bars: 50 µm (B,D), 20 µm (C).

*dGPHR* mutant larvae present a reduced growth rate and a developmental delay, which are hallmarks of mutants affecting the Insulin signaling (IS) pathway (Hietakangas and Cohen, 2009). To investigate whether *dGPHR* is implicated in IS, we analyzed the expression of two known transcriptional targets of the IS pathway in *dGPHR* mutants, namely *4E-BP* (Karim and Thummel, 1991), encoding a translational repressor and *InR*, encoding the Insulin receptor (Puig and Tjian, 2005). Both *4E-BP* and *InR* transcription is upregulated in response to repressed Insulin signaling. We found that transcription of *4E-BP* and *InR* mRNAs is unaffected in first instar larvae mutant for *dGPHR* (Fig. 3A). This suggests that *dGPHR* does not directly act in the IS pathway. However, as expected when larval growth is reduced, *dGPHR* mutants displayed reduced Insulin and Ecdysone levels (measured by the transcriptional rate of *E74B*, a direct target of the Ecdysone receptor), signaling activities in later stages of larval development (supplementary material Fig. S1). To further confirm that *dGPHR* does not act via the Insulin pathway, we followed the progeny of *dGPHR* mutant mitotic clones in wing imaginal disc. Indeed, previous reports indicated that cells defective in IS pathway component present defective growth rate and hence give rise to smaller clones than wild-type cells (Hietakangas and Cohen, 2009). As shown in Fig. 3B, *dGPHR* mutant cells grew and divided as well as neighboring cells giving rise to clones of similar size than the one derived from wild-type cells. Since imaginal cells can compensate their reduced division rate by increasing cell size, we compared the size of *dGPHR* mutant and wild-type cells by phalloidin staining and found no major difference (Fig. 3C). It is known that larval growth is largely based on an increase in cell size, which is accomplished by endoreplication, a modified cell cycle, consisting of successive rounds of DNA synthesis without intervening mitoses. We thus investigated the requirement of *dGPHR* in endoreplication by analyzing the nuclei size of *dGPHR* mutant cells induced during embryogenesis and observing in late third instar larvae (120 hr AED). Although *dGPHR* mutant larvae (120 hr AED) display salivary gland cells with reduced nuclei size when compared to controls (supplementary material Fig. S2), no obvious differences in nuclei size were noticed between *dGPHR* mutant cells and the wild-type cells, in salivary glands with MARCM clones mutant for *dGPHR* (Fig. 3D). Altogether, our data indicate that *dGPHR* does not directly act via the Insulin signaling pathway or in the endoreplication process, which are both critical regulators of larval growth. Moreover, by using a food intake assay, such as feeding with colored yeast (data not shown), analyzing metabolic markers (supplementary material Fig. S3A) or quantifying mouth hook contractions (supplementary material Fig. S3B), we could exclude the hypothesis that the smaller sizes of *dGPHR* larvae reflect the inability of *dGPHR* larvae to feed properly.

### *dGPHR* protein localizes to the endoplasmic reticulum and to the Golgi

**Fig. 4.**
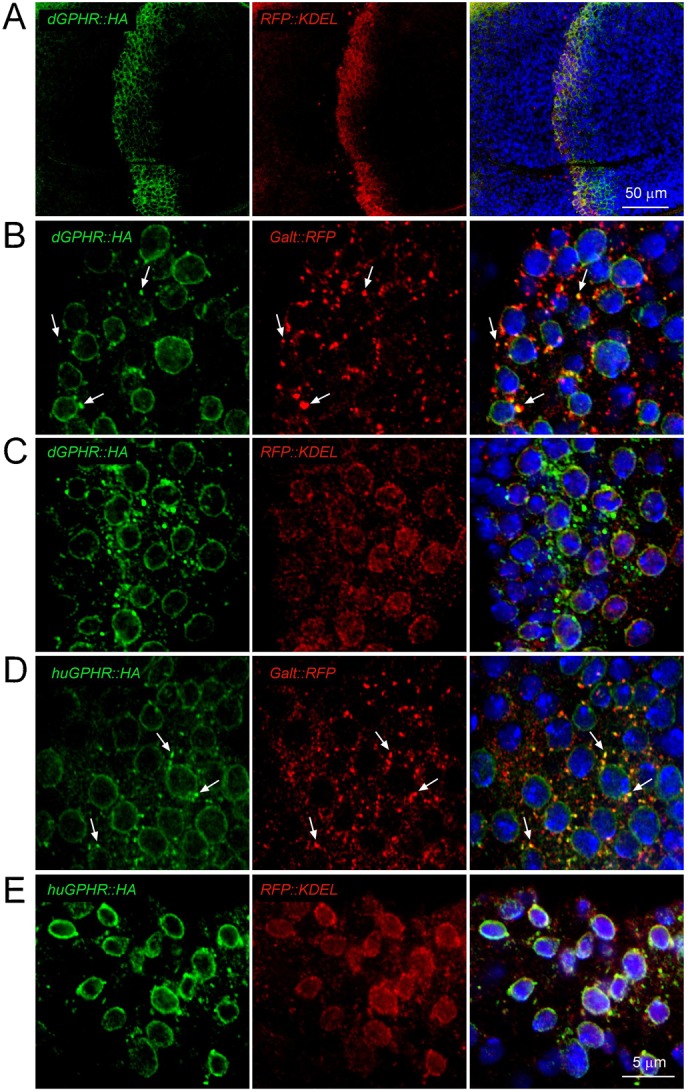
GPHR is associated with ER and Golgi markers. (A) Wing pouch region of a *patched-Gal4, UAS-dGPHR::HA; UAS-RFP::KDEL* imaginal disc showing that the dGPHR::HA and RFP::KDEL transgenes are co-expressed along the antero-posterior boundary. (B) Wing cells of a *patched-Gal4, UAS-dGPHR::HA; UAS-Galt::KDEL* larvae showing that the ponctiform GPHR-HA staining co-localized with Golgi marker. (C) Wing cells of a *patched-Gal4, UAS-dGPHR::HA; UAS-RFP::KDEL* larvae showing that the ring-like GPHR-HA staining is colocalized with ER marker. (D,E) Same experiments as in panels B and C, respectively, but with human GPHR. Scale bars: 50 µm (A), 5 µm (B–E).

The vertebrate GPHR protein has been shown to be localized and to function in the Golgi apparatus (Maeda et al., 2008). In order to test whether its invertebrate ortholog has a similar sub-cellular localization, we overexpressed an HA-tagged version of the dGPHR via the Gal4-UAS system. When expressed in a stripe of cells along the antero-posterior boundary of the wing imaginal disc using the *patched-Gal4* driver (Fig. 4A), dGPHR-HA was detectable in two distinct subcellular patterns, one surrounding the nucleus and the other inside the cytoplasm and more punctiform (Fig. 4B,C). Double labeling with specific organelle markers demonstrated that the ring-like staining largely co-localized with the ER marker RFP::KDEL and the punctiform one to a fraction of the Golgi apparatus (labeled with Galt::RFP) (Okajima et al., 2005). Similar expression patterns were observed when the human GPHR was expressed in wing disc cells (Fig. 4D,E).

### The vertebrate GPHR proteins are the functional orthologs of dGPHR

**Fig. 5.**
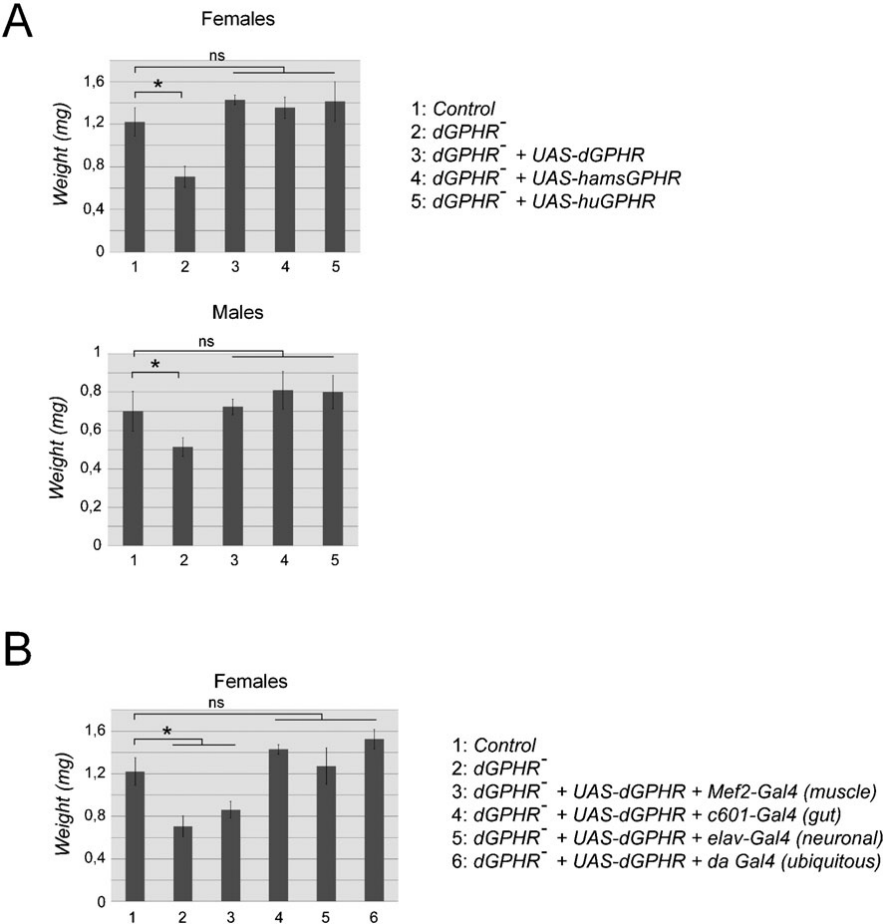
The dGPHR is the functional ortholog of the mammalian GPHR. Rescue experiment of the *dGPHR* mutant phenotype through ubiquitous (A) or tissue specific (B) overexpression of the *Drosophila* and mammalian GPHR cDNAs. The hamster and human GPHR cDNAs are as efficient as the *Drosophila* ortholog to rescue the growth defects observed in *dGPHR* mutant flies (A). This indicates that the vertebrate and invertebrate orthologs are functionally equivalent. (B) Whereas overexpression of the dGPHR cDNA in muscle is not sufficient to rescue dGPHR mutant growth defects, gut or neuronal overexpressions are. Values indicated by * are statistically significant (p<0.05).

The differential subcellular localization of GPHR in human and *Drosophila* cells prompted us to test the functional relationship between the vertebrate and invertebrate GPHRs. For that, we asked whether the vertebrate GPHR protein could functionally compensate for the lack of *dGPHR* function in flies. To do so, the human or the hamster GPHR proteins were expressed a in *dGPHR* mutant flies using a ubiquitous Gal4 driver (*daughterless-Gal4*) and the weight of the emerging adult flies was used as a readout of body size. As shown in Fig. 5A, expression of the dGPHR protein or its two vertebrate orthologs was sufficient to fully rescue *dGPHR* mutant growth defects and developmental lethality (data not shown). This indicated that the vertebrate GPHRs can compensate for a lack of dGPHR and that these proteins are therefore functional orthologs. Although *dGPHR* is predicted to be ubiquitously expressed (Flyatlas, http://flyatlas.org), we decided to investigate in which tissue *dGPHR* function was required to ensure optimal growth during *Drosophila* development. We took advantage of the ability of *UAS-dGPHR* transgene to rescue the growth defect of dGPHR mutants, and used several tissue specific Gal4 lines in rescue experiments. When expressed either in neurons using *elav-Gal4* or in gut using *c601-Gal4* or *NP1-Gal4* (data not shown) *dGPHR* could rescue the *dGPHR* mutant growth defects (Fig. 5B). In contrast, ectopic expression of *UAS-dGPHR* in muscles using *Mef2-Gal4* (Fig. 5B), in trachea using *Breathless-Gal4* or in salivary glands using *Salivary gland secretion 3-Gal4* did not (data not shown).

### *dGPHR* inactivation affects both ER and Golgi organization and impairs protein secretion

**Fig. 6.**
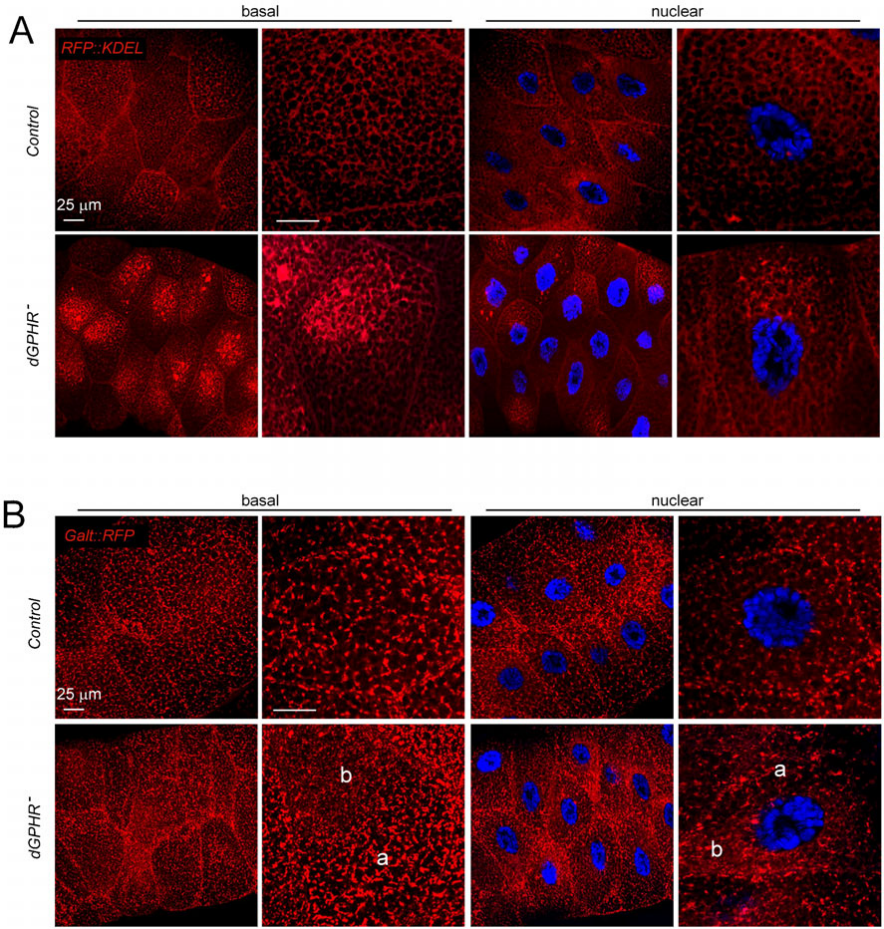
dGPHR inactivation induces ER and Golgi disorganization. Wild-type and *dGPHR* mutant salivary gland cells carrying an endoplasmic reticulum marker (RFP::KDEL, A), a Golgi marker (Galt::RFP, B). (A) The RFP::KDEL marker is uniform in control cells and concentrated both basally and nearby the nuclei in mutant cells. (B) The Galt::RFP expression pattern is form a regular network in control cells. In mutant cells, Galt::RFP staining is heterogeneous within the cells. In some regions, such as region ‘a’, Galt::RFP expression is similar to wild type, whereas in region ‘b’ it is more fragmented and weaker. This indicates that dGPHR inactivation is affecting organization of both ER and Golgi. Scale bars: 25 µm.

**Fig. 7.**
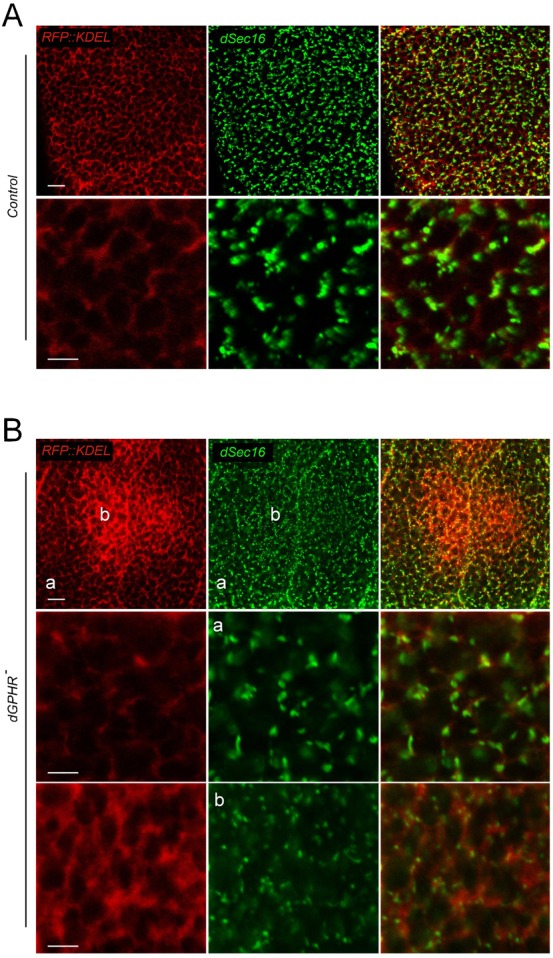
tER sites are affected in *dPGHR* mutants. Wild-type (A) and *dGPHR* mutant (B) salivary gland cells stained with anti-sec16 antibodies that detect tER sites. In mutant cells, the region in which the ER is disrupted (b) presents an abnormal pattern of sec16 expression. The tER seems more numerous but of smaller size in region ‘b’ than in region ‘a’ or than in control cells. Scale bar: 25 µm.

We then tested whether dGPHR inactivation could have effects on ER and Golgi organization. Using Galt::RFP and RFP::KDEL as Golgi and ER markers, respectively, we analyzed salivary glands of third instar larvae at 96 hr AED (a time window that precedes the growth defect phenotype of *dGPHR* mutants, Fig. 2D) and demonstrate that dGPHR inactivation was associated with severe ER and Golgi disorganization phenotypes (Fig. 6A,B). Whereas RFP::KDEL forms a uniform network in wild-type salivary gland cells, it accumulates basally and asymmetrically in the center of the cell in proximity to the nucleus in mutant tissue (Fig. 6A). The global organization of the Golgi apparatus was also affected by dGPHR inactivation. The regular dot-like pattern detected in wild-type cells carrying a Galt::RFP transgene was disrupted in mutant cells although not uniformly. Whereas in some regions of the mutant cells (labeled ‘a’ in Fig. 6B), Golgi structure appears similar to control, in others (labeled ‘b’), Galt::RFP staining appears more fragmented and fainter than in controls. To insure that these effects were not an indirect consequence of the *dGPHR* mutation on growth retardation (i.e. on Insulin signaling reduction), we tested whether similar phenotypes can be observed in *chico^1^* mutant salivary glands (at 96 hr (data not shown) and 120 hr AED). As shown in supplementary material Fig. S4, *chico^1^* salivary gland cells present a homogeneous distribution of both RFP::KDEL and Galt::RFP, similar to wild-type glands. To further characterize the defaults observed in organelles associated with the secretory pathway, wild-type and mutant cells were labeled with the ER exit site marker sec16. As already observed with Golgi and ER markers, the distribution of sec16 positive structures was quite heterogeneous in mutant cells. Whereas in some compartments of mutant cells, tER sites were uniformly scattered and spaced (labeled ‘a’ in Fig. 7B) as in wild-type controls, in others domains (labeled ‘b’) the sec16 positive structures were more fragmented (Fig. 7B). The cell compartments in which sec16 straining was fragmented correspond to domains in which RFP::KDEL was abnormally accumulated (Fig. 7B). This disorganization of ER was confirmed using electron microscopy. Whereas TEM pictures of wild-type salivary gland cells present large vesicles of secretion surrounded with well-organized ER ribbon, the cytoplasmic organization of *dGPHR* mutant cells was strikingly different (supplementary material Fig. S5). The size of secretory granules was 5 times smaller in mutant than in wild-type cells. In addition, the very well-organized ER ribbon with associated ribosomes seen in wild-type cells was no longer detectable in *dGPHR* mutant cells. The abnormal ER organization associated with dGPHR mutation, led us to test the functionality of secretory pathway. For this purpose, we make use of a sec::GFP reporter construct that allows us to visualize the secretory process in vivo. In contrast to control cells in which the sec::GFP protein is present throughout the cytoplasm in a uniform pattern, its expression pattern was very similar to that of the ER markers in *dGPHR* mutant cells (supplementary material Fig. S6). Altogether, these data indicate that in the absence of functional dGPHR, both the Golgi apparatus and the ER are disorganized indicating that the dGPHR protein is required to shape the ER-Golgi apparatus and that its inactivation affect the secretory pathway functionality.

## Discussion

We report here the first analysis of a Golgi pH Regulator mutant in *Drosophila melanogaster*. In contrast to what is expected for a loss-of-function mutation in a gene thought to be involved in fundamental cellular processes, complete removal of maternal (data not shown) and zygotic dGPHR function is not fully lethal and can generate viable flies. This is rather unexpected knowing that functional redundancy is relative rare in flies and clearly less pronounced than in vertebrates. Although the complete mouse knockout phenotype has not been yet reported, a recent report indicate that mice lacking GPHR in keratinocytes exhibited hypo-pigmented skin, hair loss and scaliness, indicating that mammal GPHR is essential for epidermis homeostasis (Tarutani et al., 2012). One possible explanation for the relative milder phenotype in *Drosophila* could be that pH acidification is under the regulation of multiples molecules or pathways. Another possibility would be that dGPHR is not functioning as Golgi lumen acidificator. This later hypothesis is, however, unlikely as we have shown that the vertebrate GPHR can compensate for the absence of the *Drosophila* ortholog, suggesting that the two proteins are performing similar functions. It should, however, be mentioned that our results indicate that the mammalian and flies GPHR do not have the same sub-cellular localization. Whereas the vertebrate GPHR is a Golgi only associated protein, its *Drosophila* ortholog seems to be associated with both the Golgi and the ER. We cannot, however, exclude that accumulation in the ER in secondary to the overexpression of these proteins. Although this do not definitively ruled out this hypothesis, it should be noted that dGPHR::HA staining nicely colocalize with ER marker whereas it only partially overlaps with Golgi staining. The different sub-cellular localization between *Drosophila* and mammalian GPHR could also relate to different organization of the pathway in invertebrates and vertebrates. Indeed, if the molecular principles underlying the secretory pathway are largely conserved between mammals and *Drosophila*, some differences exist (Kondylis and Rabouille, 2009). Whereas the *Drosophila* secretory pathway consist of tER sites closely associated to individual Golgi stacks, its mammalian homolog structure is made of Golgi stacks linked to form a single copy organelle forming a ribbon (Rabouille et al., 1999; Ripoche et al., 1994). Further studies will be required to understand whether these differences could be explained by morphological or functional differences between flies and vertebrate secretory pathway. Although dGPHR mutant embryos can develop into adult, those are much smaller that wild-type flies indicating that Golgi function is regulating growth. In metazoans, the Insulin/Insulin-like growth factor signaling pathway controls the growth rate of tissues according to nutrient availability. Recent studies have described that communication between different larval tissues is essential to influence overall body growth and development although in many cases, the nature of the secreted factors that mediate this inter-organ signaling is still unclear. For example, *Drosophila* growth is mediated by Insulin-like peptides that are produced by specific neurons in the brain and released into the hemolymph to couple nutrient uptake with systemic growth (Colombani et al., 2003; Géminard et al., 2009). Upstream of the event is the secretion by the fat body of unknown signalling molecules that circulate in the hemolymph to reach the brain in order to control Insulin secretion (Géminard et al., 2009). In addition, animal growth relies on nutrient processing, which involves the secretion of digestive enzymes by gut cells. Hence, one putative explanation for the dwarf phenotype associated with the *dGPHR* mutation could correspond to the impairment of some cells that normally produced and release proteins that favour growth, to do so. For instance, one could imagine that the Insulin producing cells and/or the fat body, and/or the gut cells have reduced capacity in secreting such molecules when the function of dGPHR is impaired. The ability of *UAS-dGPHR* transgene to rescue the growth defect of dGPHR mutants when expressed either in neuronal cells or gut cells are in agreement with this hypothesis and suggest that restoring secretion in one tissue can compensate for defects in other tissues. Nevertheless, further work will be required to address these issues.

## Supplementary Material

Supplementary Material
