# Supplementary material for: Mutations in the Drosophila ortholog of the vertebrate Golgi pH regulator (GPHR) protein disturb endoplasmic reticulum and Golgi organization and affect systemic growth
Source: Biol Open. 2013 Dec 6;3(1):72–80. doi: 10.1242/bio.20137187 (PMC3892162; doi:10.1242/bio.20137187)
Supplement: Supplementary Material [file supp_3_1_72__index.html]

Mutations in the Drosophila ortholog of the vertebrate Golgi pH regulator (GPHR) protein disturb endoplasmic reticulum and Golgi organization and affect systemic growth — Mutations in the Drosophila ortholog of the vertebrate Golgi pH regulator (GPHR) protein disturb endoplasmic reticulum and Golgi organization and affect systemic growth — Supplementary Material 

# Mutations in the *Drosophila* ortholog of the vertebrate Golgi pH regulator (GPHR) protein disturb endoplasmic reticulum and Golgi organization and affect systemic growth

## bio.20137187 Supplementary Material

**Files in this Data Supplement:**

- Supplementary Material - Bernard Charroux and Julien Royet doi: 10.1242/bio.20137187
