# Supplementary material for: Mutations in the Drosophila ortholog of the vertebrate Golgi pH regulator (GPHR) protein disturb endoplasmic reticulum and Golgi organization and affect systemic growth
Source: Biol Open. 2013 Dec 6;3(1):72–80. doi: 10.1242/bio.20137187 (PMC3892162; doi:10.1242/bio.20137187)
Supplement: Supplementary Material [file supp_bio.20137187_bio.20137187-s1.pdf]

Supplementary Material

Bernard Charroux and Julien Royet **doi: 10.1242/bio.20137187**

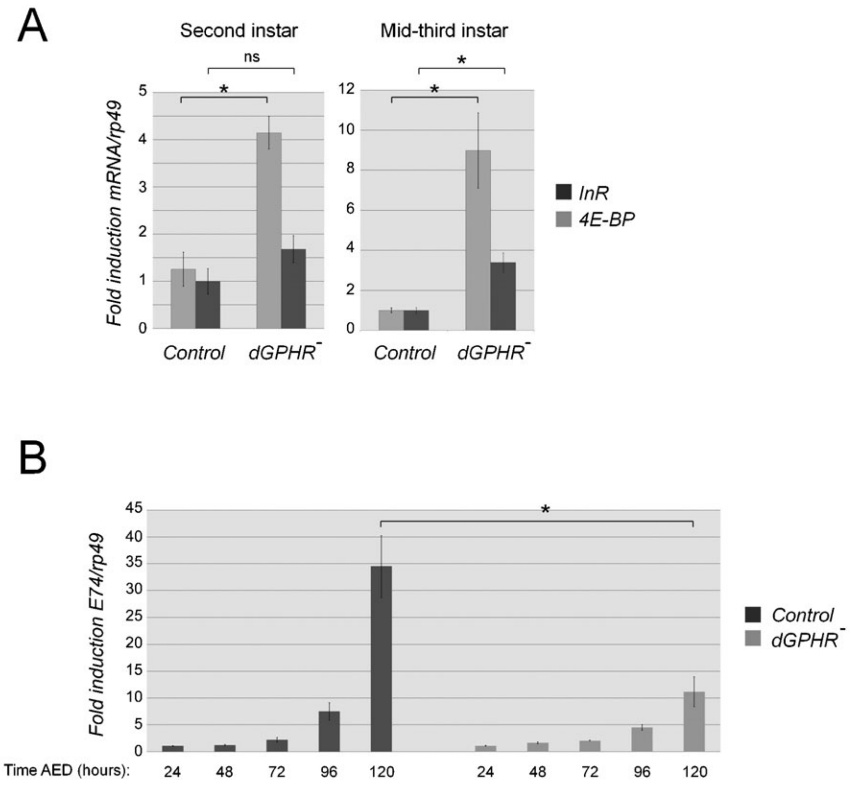

Fig. S1. *dGPHR* mutant larvae display Insulin-like phenotype at L2 and L3 stage. (A) mRNA quantification by qRT-PCR of two transcriptional targets of the IS pathway. *dGPHR* mutation affects *InR* and *4E-BP* transcription in second and third instar larvae. (B) *E74B* mRNA levels from day 2 AED to day 10 AED mutant and control larvae. Values indicated by * are statistically significant ($p<0.05$).

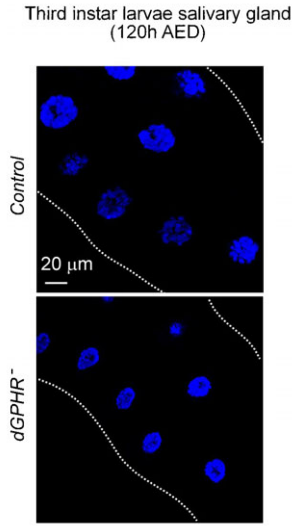

Fig. S2. Late third instar larvae mutant for *dGPHR* have reduced nuclei size. Confocal microscope section of salivary gland cell nuclei from controls and *dGPHR* mutant larvae at 120 hr AED are shown. Scale bar: 20 µm.

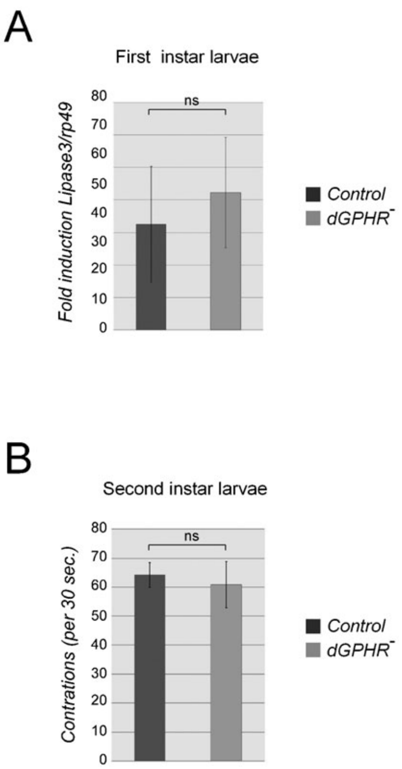

Fig. S3. Feeding behavior and *Lipase 3* transcription is normal in *dGPHR* mutants. (A) mRNA quantification by qRT-PCR of the metabolic marker *Lipase 3*. *dGPHR* mutation do not affect *Lipase 3* transcription in first instar larvae.
(B) The number of mouth hook contractions of larvae feeding in either solid glucose agar was counted in a 30 s period. No significant differences were found between controls and *dGPHR* mutants.

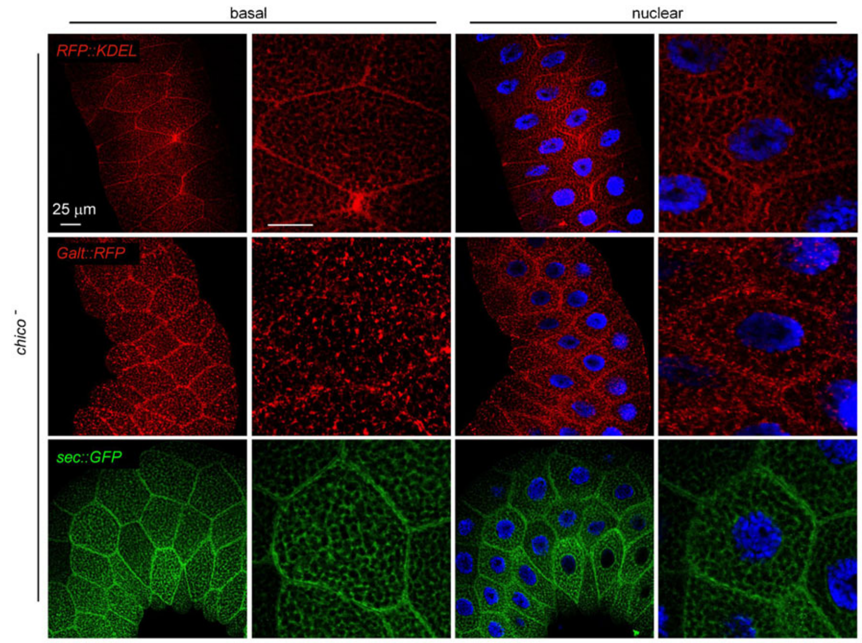

Fig. S4. Insulin pathway mutant cells have normal Golgi and ER. Salivary gland cells from *chico*^{1} mutant have a Golgi and an ER organization indistinguishable from wild-type controls. Scale bars: 25 µm.

Biology Open

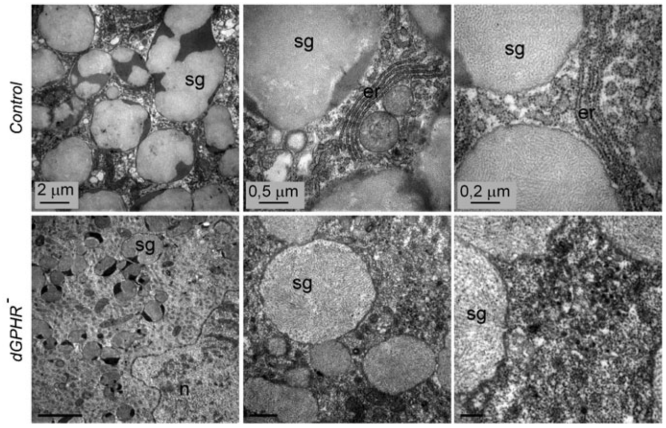

Fig. S5. TEM images of control and *dGPHR* mutant cells. Salivary gland cells from wild type and *dGPHR* mutant were analyzed by TEM. Secretory granules (SG) are much smaller in mutant than in wild-type cells. Whereas classical endoplasmic reticulum (ER) structures with saccules surrounded by ribosomes are present in between secretory granules, these structures are not seen in the mutant cells. n: nucleus. Scale bars: 2 µm (left), 0.5 µm (middle), 0.2 µm (right).

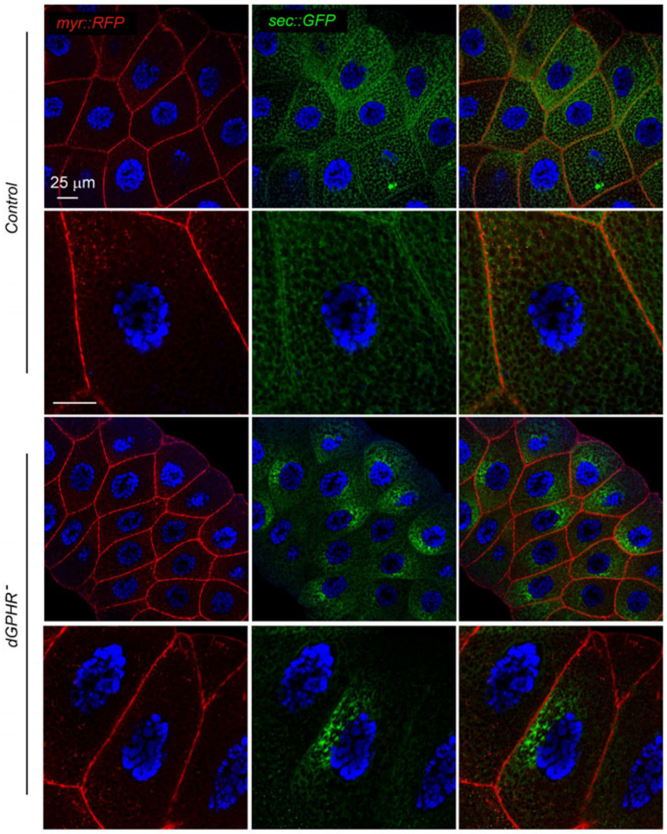

Fig. S6. Sec::GFP pattern in control and *dGPHR* mutant cells. Salivary gland cells carrying a sec::GFP reporter construct, which labels the secretory pathway. Sec::GFP marker is uniform in control cells and concentrated asymmetrically in the cytoplasm and nearby the nuclei of mutant cells. Scale bars: 25 µm.
